# Icariside II activates EGFR-Akt-Nrf2 signaling and protects osteoblasts from dexamethasone

**DOI:** 10.18632/oncotarget.13732

**Published:** 2016-12-01

**Authors:** Weidong Liu, Li Mao, Feng Ji, Fengli Chen, Shouguo Wang, Yue Xie

**Affiliations:** ^1^ Department of Orthopedics, Huai’an First People's Hospital, Nanjing Medical University, Huai’an, China; ^2^ Department of Endocrinology, Huai’an First People's Hospital, Nanjing Medical University, Huai’an, China; ^3^ Clinical Laboratory, Huai’an First People's Hospital, Nanjing Medical University, Huai’an, China

**Keywords:** icariside II, dexamethasone, EGFR-Akt, Nrf2 signaling, oxidative stress

## Abstract

The potential effect of icariside II on dexamethasone-induced osteoblast cell damages was evaluated here. In MC3T3-E1 osteoblastic cells and the primary murine osteoblasts, co-treatment with icariside II dramatically attenuated dexamethasone- induced cell death and apoptosis. Icariside II activated Akt signaling, which is required for its actions in osteoblasts. Akt inhibitors (LY294002, perifosine and MK-2206) almost abolished icariside II-induced osteoblast cytoprotection against dexamethasone. Further studies showed that icariside II activated Nrf2 signaling, downstream of Akt, to inhibit dexamethasone-induced reactive oxygen species (ROS) production in MC3T3-E1 cells and primary osteoblasts. On the other hand, Nrf2 shRNA knockdown inhibited icariside II-induced anti-dexamethasone cytoprotection in MC3T3-E1 cells. Finally, we showed that icariside II induced heparin-binding EGF (HB-EGF) production and EGFR trans-activation in MC3T3-E1 cells. EGFR inhibition, via anti-HB-EGF antibody, EGFR inhibitor AG1478 or EGFR shRNA knockdown, almost blocked icariside II-induced Akt-Nrf2 activation in MC3T3-E1 cells. Collectively, we conclude that icariside II activates EGFR-Akt-Nrf2 signaling and protects osteoblasts from dexamethasone. Icariside II might have translational value for the treatment of dexamethasone-associated osteoporosis/osteonecrosis.

## INTRODUCTION

Dexamethasone (Dex) is a common anti-inflammatory medicine that is utilized by a huge number of patients [1]. Yet, excessive Dex application could cause secondary osteoporosis [2, 3] and/or osteonecrosis [4]. Studies have shown that about one third of patients with long-term Dex treatment could suffer bone damages [2]. Osteoblast cell apoptosis was often observed in bones of the Dex-using patients [2–4]. *In vitro*, exogenously-added Dex could induce direct damages to the cultured osteoblastic cells and osteoblasts [5–10]. The research focus of our group [5–7] is to explore the signaling mechanisms of Dex-induced osteoblast cell death, and to develop potential intervention strategies.

Icariside II is one of the three main active components and a natural glycoside derived from Chinese medicine *E. Koreanum* [11, 12]. Existing evidences have shown that icariside II could exert a number of biological functions under a number of experimental settings. For example, several studies have confirmed the pro-survival function of icariside II [13, 14]. Others, however, showed that icariside II may induce apoptosis in number of cancer cells [12, 15–17]. Whether icariside II could protect Dex- induced osteoblast cell damages has not been studied. And if so, the underlying signaling mechanisms are largely unknown. In the current study, we show that icariside II protects osteoblasts from Dex via activating epidermal growth factor receptor (EGFR)-Akt-NF-E2-related factor 2 (Nrf2) signaling.

## RESULTS

### Icariside II protects osteoblasts from Dex

**Figure 1:**
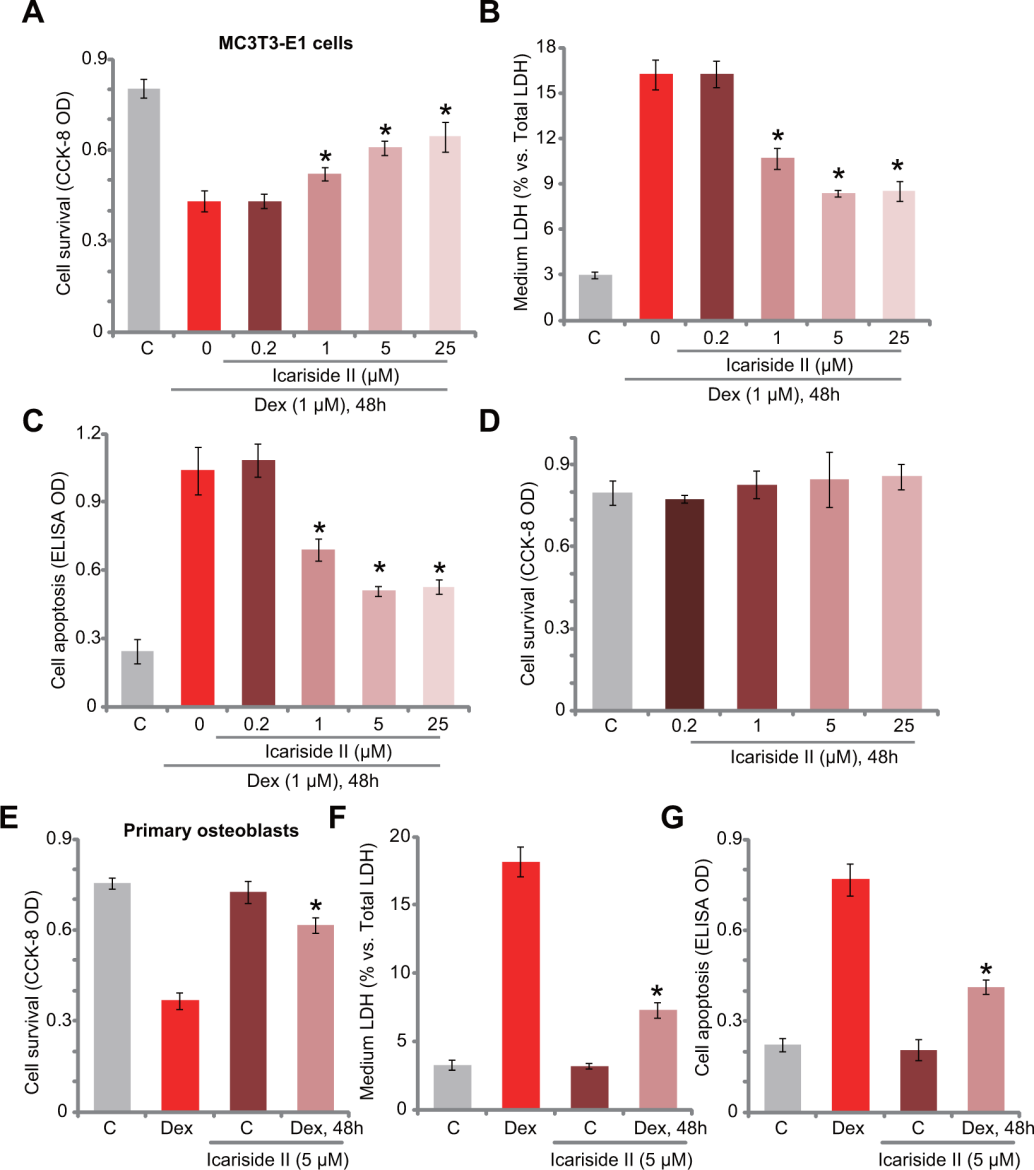
Icariside II protects osteoblasts from Dex MC3T3-E1 osteoblastic cells (**A**–**D**) or the primary murine osteoblasts (**E**–**G**) were treated with dexamethasone (“Dex”, 1 μM) and/or indicated concentration of icariside II (0.2–25 μM), cells were then cultured in conditional medium for 48 hours; Cell survival was tested by the CCK-8 assay (A, D and E); Cell death was examined by the LDH release assay (B and F); Cell apoptosis was tested by the Histone DNA ELISA assay (C and G). Data are shown as mean (*n* = 5) ± standard deviation (SD) (Same for all figures). “C” stands for untreated control (Same for all figures). Experiments in this figure were repeated for a total of five times, and similar results were obtained each time. *P < 0.05 vs. Dex only group.

In order to study the potential effect of icariside II on Dex-induced cytotoxicity in osteoblasts, MC3T3-E1 osteoblastic cells were treated with Dex (1 μM) or plus gradually increased concentration of icariside II (0.2–25 μM). Results demonstrated that icariside II, at 1–25 μM, significantly attenuated Dex-induced MC3T3-E1 cell viability reduction (CCK-8 assay, Figure 1A), cell death (LDH release assay, Figure 1B) and apoptosis (Histone DNA ELISA assay, Figure 1C). Icariside II demonstrated a dose-dependent activity in protecting MC3T3-E1 cells from Dex (Figure 1A–1C). Of the tested concentrations, 5 μM of icariside II efficiently inhibited Dex-induced cytotoxicity. This concentration was chosen for the following mechanistic studies. Treatment with icariside II (0.2–25 μM) alone failed to inhibit MC3T3-E1 cell survival (Figure 1D). We also tested icariside II's activity in the primary murine osteoblasts. As demonstrated, co-treatment with 5 μM of icariside II in the primary murine osteoblasts dramatically attenuated Dex (1 μM)-induced cell viability reduction (Figure 1E), cell death (Figure 1F) and apoptosis (Figure 1G). Icariside II alone again failed to inhibit survival of osteoblast cells (Figure 1E–1G). The results of these study clearly demonstrate that icariside II protects osteoblastic cells and primary osteoblasts from Dex.

### Akt activation is required for icariside II-induced osteoblast cytoprotection against Dex

**Figure 2:**
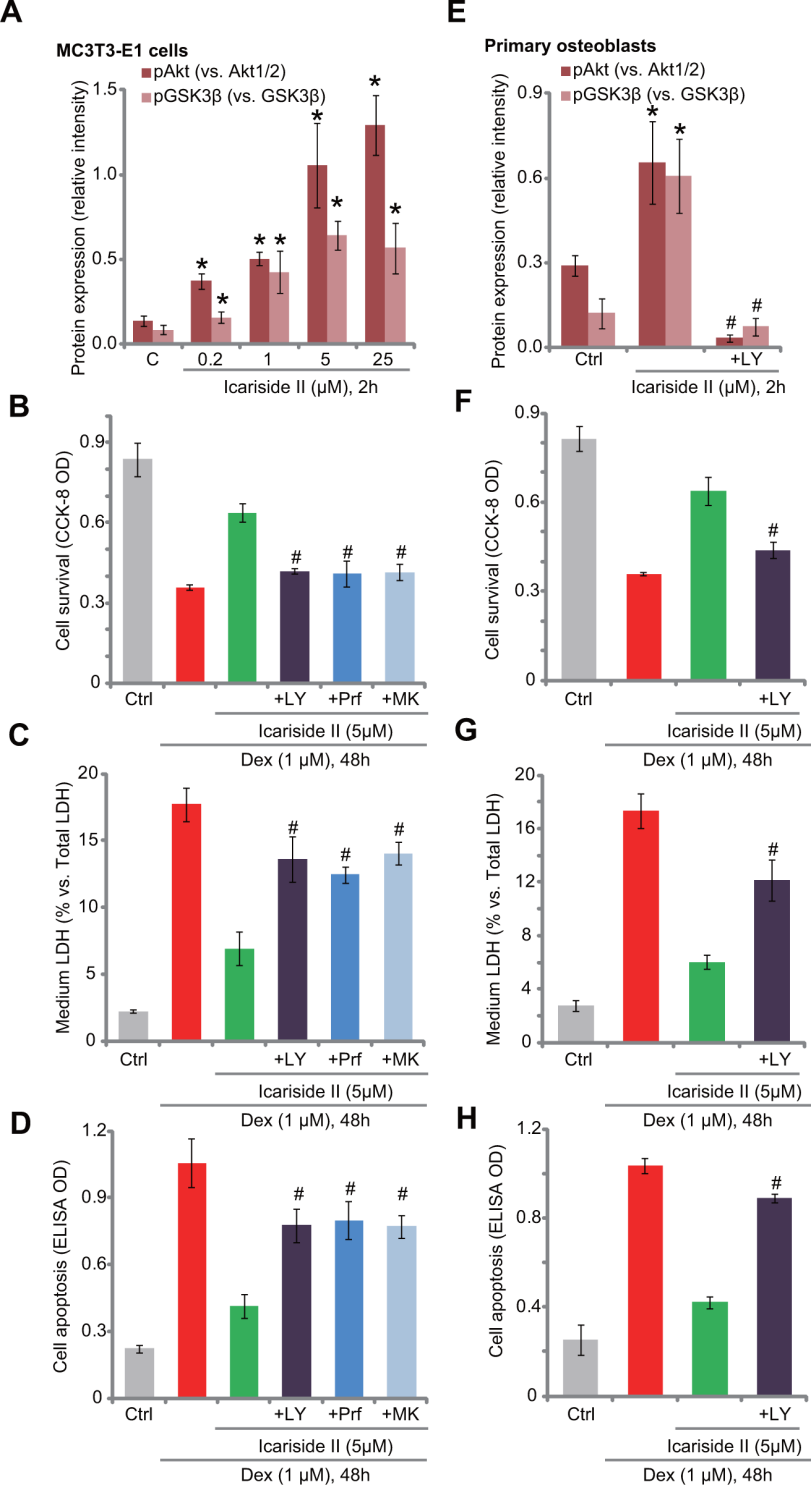
Akt activation is required for icariside II-induced osteoblast cytoprotection against Dex Expressions of Akt and GSK3β (phosphorylated and regular) in MC3T3-E1 osteoblastic cells and primary murine osteoblasts with designated treatment were tested by Western blot assay, and results of three repeats were quantified (**A** and **E**). MC3T3-E1 osteoblastic cells (**B**–**D**) or primary murine osteoblasts (**F**–**H**) were pre-treated for 30 min with listed Akt inhibitor: LY294002 (“+LY”, 500 nM), MK-2206 (“+MK”, 5 μM) or perifosine (“+Prf” , 5 μM), followed by icariside II (5 μM) and dexamethasone (“Dex”, 1 μM) treatment, cells were then cultured in conditional medium for 48 hours; Cell survival was tested by the CCK-8 assay (B and F); Cell death was examined by the LDH release assay (C and G); Cell apoptosis was tested by the Histone DNA ELISA assay (D and H). Experiments in this figure were repeated for three times, and similar results were obtained each time. *P < 0.05 vs. “C” group. ^#^*P <* 0.05 vs. icariside II only group.

Studies including ours [6, 7, 10, 18] have shown that activation of Akt, a major pro-survival signaling [19], could efficiently protect osteoblasts from Dex. We thus analyzed Akt signaling in icariside II-treated cells. Western blot assay results in Figure 2A demonstrated that icariside II treatment in MC3T3-E1 osteoblastic cells dose-dependently induced Akt activation, the latter was evidenced by phosphorylation of Akt (at Ser-473) and its major downstream GSK3β (at Ser-9) (see quantified results in Figure 2A). The expression of regular Akt1/2 and GSK3β was unchanged following the same icariside II treatment. To study the potential effect of Akt activation in icariside II-induced osteoblast cytoprotection against Dex, a panel of Akt inhibitors were applied, including the PI3K- Akt pan inhibitor LY294002, as well as the two Akt specific inhibitors perifosine [20] and MK-2206 [21]. As expected, treatment with these Akt inhibitors (LY294002, perifosine and MK-2206) blocked Akt activation by icariside II (5 μM) (Data not shown). More importantly, icariside II-induced anti-Dex cytoprotection was almost abolished with the presence of the Akt inhibitors in MC3T3-E1 cells (Figure 2B–2D). In the other words, icariside II was no longer effective or cytoprotective against Dex when Akt was blocked (Figure 2B–2D). In the primary murine osteoblasts, icariside II (5 μM)-induced Akt activation, or Akt-GSK3β phosphorylation, was also blocked by LY294002 (see quantified results in Figure 2E). The latter almost abolished icariside II-induced anti-Dex actions in the primary cells (Figure 2F–2H). These results suggest that activation of Akt is required for icariside II-induced osteoblast cytoprotection against Dex.

### Icariside II activates Nrf2 signaling and inhibits Dex-induced ROS production

**Figure 3:**
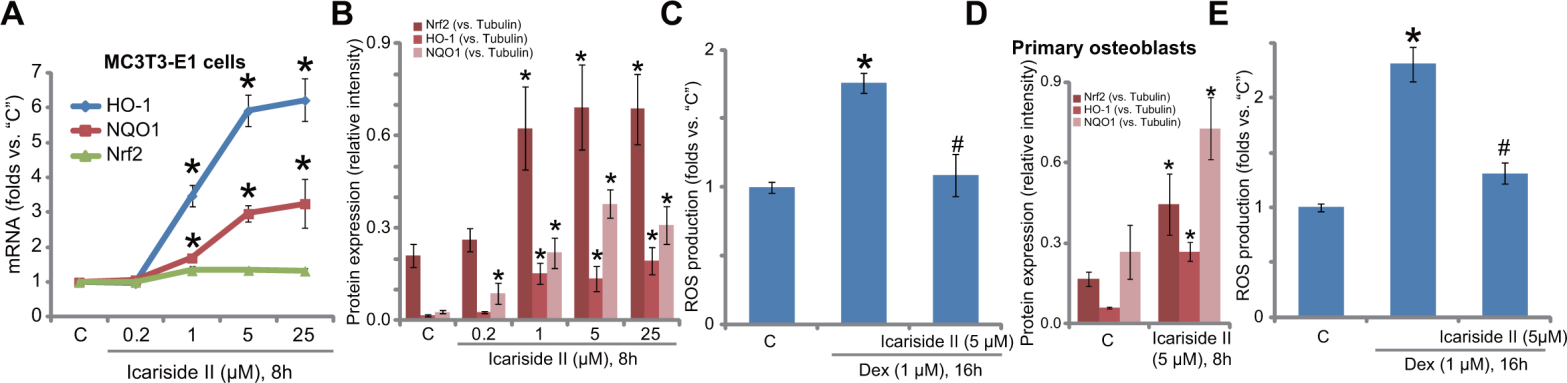
Icariside II activates Nrf2 signaling and inhibits Dex-induced ROS production MC3T3-E1 osteoblastic cells (**A**–**C**) or the primary murine osteoblasts (**D**–**E**) were treated with indicated concentration of icariside II (0.2–25μM), or plus dexamethasone (“Dex”, 1 μM), cells were cultured in the conditional medium for indicated time; mRNA (A) and protein (B and D, results of three repeats were quantified) expression of listed genes were shown; Relative ROS intensity was tested by the DCFH-DA fluorescent assay (C and E). Experiments in this figure were repeated for a total of three times, and similar results were obtained each time. *P < 0.05 vs. “C” group. ^#^*P <* 0.05 vs. Dex only group.

Growing evidences have shown that Dex induces reactive oxygen species (ROS) production and oxidative stress in osteoblasts, which cause subsequent cell damages [5, 22]. On the other hand, ROS inhibition could protect osteoblasts from Dex [22–24]. NF-E2-related factor 2 (Nrf2) signaling is a well-defined and potent anti-oxidant signaling [25, 26], and recent studies have proposed that Akt activation could function as a Nrf2 upstream signaling [27–29]. Therefore, Nrf2 signaling in icariside II-treated cells was tested here. Real-time quantitative PCR (RT-qPCR) assay results in Figure 3A demonstrated that icariside II dose-dependently increased transcription or mRNA expression of Nrf2-dictated genes, including *heme oxygenase-1 (HO-1)* and *NAD [P] H: quinone oxidoreductase 1 (NQO1)* [5, 22] in MC3T3-E1 cells. HO-1 and NQO1 protein expression was also increased with icariside II (1–25 μM) treatment (see quantified results in Figure 3B). Interestingly, Nrf2 mRNA was unchanged following the same icariside II treatment in MC3T3-E1 cells (Figure 3A). However, Nrf2 protein level was significantly increased (see quantified results in Figure 3B). These results indicate that icariside II induced Nrf2 stabilization, a necessary step for its activation [5, 22], which likely promoted transcription of Nrf2-dictated genes (*HO-1* and *NQO1*). Remarkably, icariside II (5 μM) significantly inhibited Dex-induced ROS production in MC3T3-E1 cells (Figure 3C). Very similar results were also achieved in the primary murine osteoblasts, where icariside II (5 μM) induced protein upregulation of Nrf2, HO-1 and NQO1 (see quantified results in Figure 3D) and inhibited Dex-induced oxidative stress (Figure 3E).

### Nrf2 activation is required for icariside II-mediated anti-Dex actions in MC3T3-E1 cells

**Figure 4:**
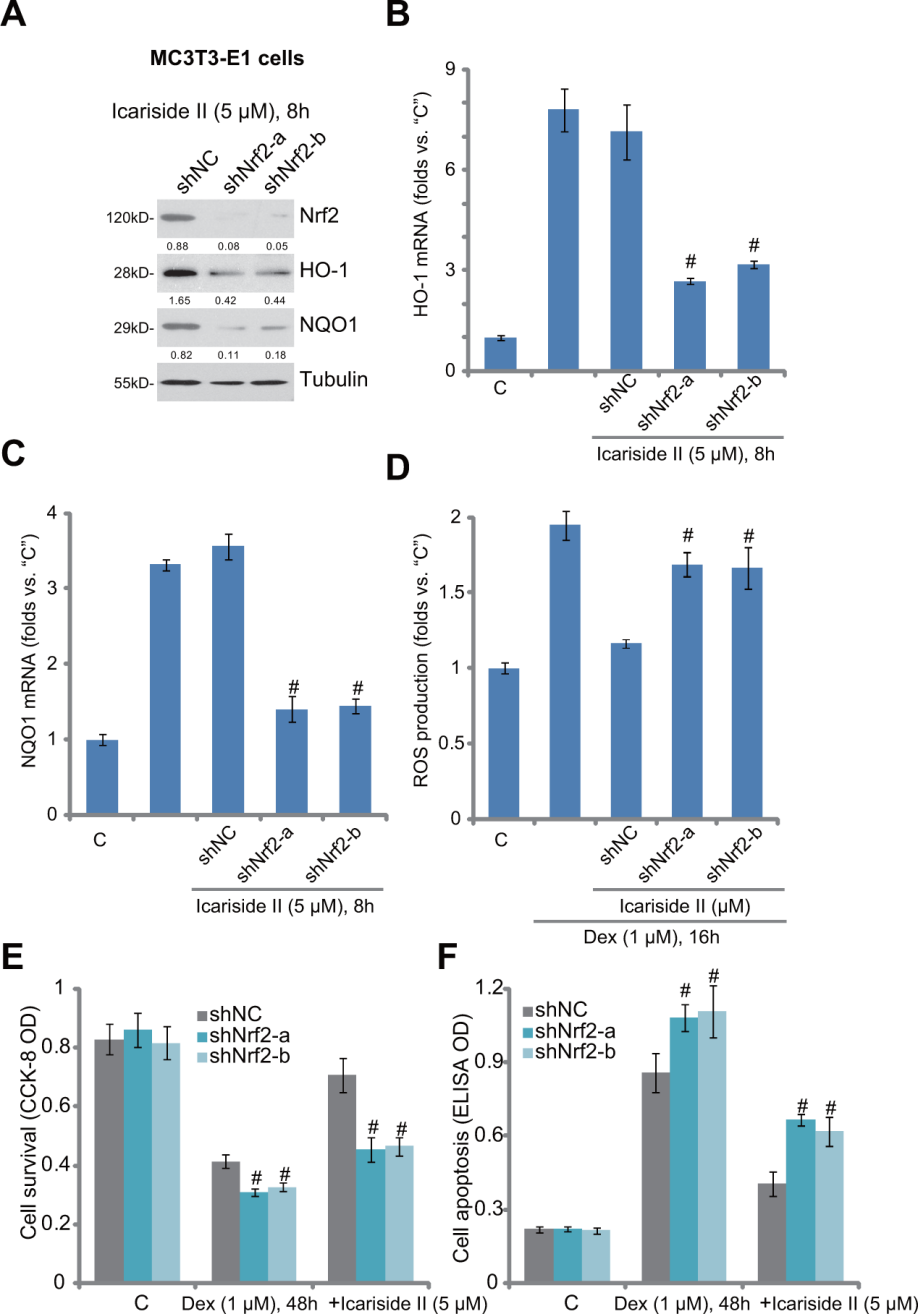
Nrf2 activation is required for icariside II-mediated anti-Dex actions in MC3T3-E1 cells Puromycin-selected stable MC3T3-E1 cells, expressing non-sense control shRNA (“shNC”) or Nrf2 shRNA (“shNrf2-a or -b”), were treated with icariside II (5 μM) or plus dexamethasone (“Dex”, 1 μM), cells were further cultured in the conditional medium for applied time; Protein (**A**) and mRNA (**B** and **C**) expression of listed genes were shown; Relative ROS content was analyzed by the DCFH-DA fluorescent assay (**D**); Cell survival and apoptosis were tested by the CCK-8 assay (**E**) and Histone DNA apoptosis ELISA assay (**F**), respectively. Relative expression of listed proteins (vs. Tubulin) were quantified (A). Experiments in this figure were repeated for three times, and similar results were obtained each time. ^#^*P <* 0.05 vs. “shNC” group.

The above results showed that icariside II activated Nrf2 signaling and inhibited Dex-induced cytotoxicity in osteoblasts. To further explore the link between the two, we applied shRNA method to knockdown Nrf2 in MC3T3-E1 cells. Western blot assay results in Figure 4A demonstrated that the two applied Nrf2 shRNAs (“shNrf2−a/−b”, with non-overlapping sequences) potently downregulated Nrf2 in MC3T3-E1 cells. Consequently, icariside II (5 μM)-induced protein expression of HO-1 and NQO1 was also inhibited (Figure 4A). Meanwhile, *HO-1* and *NQO1* mRNA expression in icariside II-treated MC3T3-E1 cells was almost blocked with Nrf2 silence (Figure 4B and 4C). More importantly, icariside II (5 μM)-induced ROS scavenging ability in Dex-treated MC3T3-E1 cells was almost abolished after Nrf2 shRNA knockdown (Figure 4D). These results confirm that icariside II-induced anti-oxidant activity indeed requires Nrf2 activation.

Next, we tested whether Nrf2 activation was also important for icariside II-mediated cytoprotection against Dex. We showed that icariside II (5 μM)-induced pro-survival (Figure 4E) and anti-apoptosis (Figure 4F) activities against Dex were largely inhibited in Nrf2-silneced MC3T3-E1 cells. Thus, icariside II-mediated osteoblast cytoprotection was largely compromised with Nrf2 knockdown (Figure 4E and 4F). Intriguingly, when Nrf2 was silenced, Dex-induced cytotoxicity and apoptosis were also slightly but significantly increased in MC3T3-E1 cells (Figure 4E and 4F), indicating that basal Nrf2 activation is also important for Dex-resistance in osteoblastic cells.

### EGFR trans-activation mediates icariside II-induced Akt and Nrf2 activation in osteoblasts

**Figure 5:**
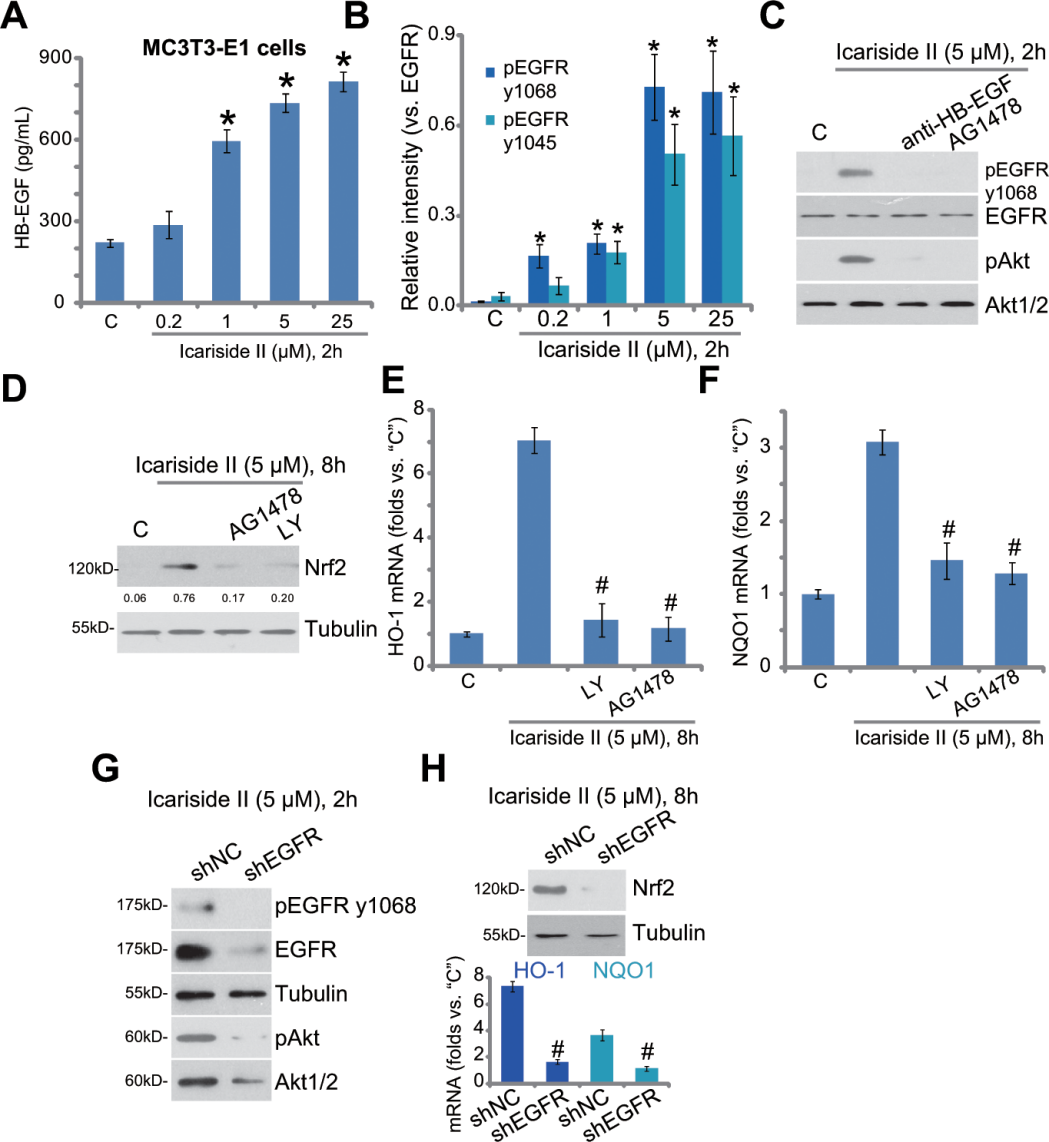
EGFR trans-activation mediates icariside II-induced Akt and Nrf2 activation in osteoblasts MC3T3-E1 cells were treated with indicated concentration of icariside II (0.2–25 μM), cells were then cultured for indicated time; HB-EGF content in the conditional medium was evaluated by the ELISA assay (**A**); Expression of listed proteins was tested by the Western blot assay (**B**, results of three repeats were quantified). MC3T3-E1 cells, pretreated for 30 min with anti-HB-EGF (100 μg/mL), AG1478 (500 nM) or LY294001 (“LY”, 500 nM), were treated with icariside II (5 μM), cells were further cultured for designated time; Expression of listed proteins was shown (**C** and **D**); mRNA expression of *HO-1* (**E**) and *NQO1* (**F**) was also tested. Puromycin-selected MC3T3-E1 cells, expressing non-sense control shRNA (“shNC”) or EGFR shRNA (“shEGFR”), were treated with icariside II (5 μM) for indicated time; Protein or mRNA expression of listed genes were shown (**G** and **H**). Experiments in this figure were repeated for three times, and similar results were obtained each time. *P < 0.05 vs. “C” group. ^#^*P <* 0.05 vs. icariside II only group.

At last, we tested the possible upstream signaling for icariside II-induced Akt-Nrf2 activation. Several very recent studies have proposed that activation or trans-activation of epidermal growth factor receptor (EGFR) could protect osteoblasts from Dex [10, 18]. Thus, we tested EGFR signaling in icariside II-treated MC3T3-E1 cells. We discovered that the content of HB-EGF (heparin-binding epidermal growth factor) was significantly increased following treatment of icariside II (1–25 μM) in MC3T3-E1 cells (Figure 5A). Meanwhile, Western blot assay results showed that icariside II dose- dependently increased phosphorylation of EGFR at both Tyr-1068 and Tyr-1045, indicating significant EGFR activation (Figure 5B, quantified results of three repeats) [30]. Remarkably, blockage of EGFR activation, by the EGFR inhibitor AG1478 [31] or anti-HB-EGF monoclonal antibody [18], almost abolished icariside II- induced Akt activation (Figure 5C) and Nrf2 accumulation (Figure 5D) in MC3T3-E1 cells. AG1478 and the Akt inhibitor LY294002 also inhibited *HO-1* and *NQO1* mRNA expression in icariside II-treated cells (Figure 5E and 5F), suggesting that EGFR-Akt likely serves as the upstream signaling for icariside II-induced Nrf2 signaling activation.

To further support the requirement of EGFR in icariside II-induced actions, shRNA method was again applied to stably knockdown EGFR in MC3T3-E1 cells. The applied EGFR shRNA efficiently downregulated EGFR, and inhibited activation of EGFR-Akt (Figure 5G) and Nrf2 (Figure 5H) in MC3T3-E1 cells. Collectively, these results show that EGFR trans-activation mediates icariside II-induced Akt and Nrf2 activation in osteoblasts.

## DISCUSSION

Dex could induce significant ROS production and oxidative stress in osteoblasts/osteoblastic cells, which could be a major contributor of subsequent cell death [5, 7–9, 22]. As a matter of fact, several ROS scavengers were shown to block the above process and to efficiently rescue osteoblasts from Dex [5, 7, 8, 22]. For example, Guo *et al*., showed that Compound 13 (C13) activates AMP-activated protein kinase (AMPK)-dependent signaling to inhibit Dex-induced oxidative stress and protect osteoblasts [5]. Fan *et al*., showed that microRNA- 135b (“miR-135b”) expression similarly provokes AMPK signaling to inhibit Dex-induced oxidative damages in osteoblasts [9]. In line with these findings, we show that icariside II activated Akt downstream Nrf2 signaling to inhibit Dex-induced ROS production and osteoblast cell death. On the contrary, Nrf2 shRNA knockdown largely attenuated icariside II-induced ROS clearance activity and almost abolished anti-Dex cytoprotection in osteoblasts. Therefore, Nr2 activation and ROS scavenging could be the key mechanism responsible for icariside II's pro-survival actions in osteoblasts.

Nrf2 phosphorylation at Ser-40 is required for Nrf2 stabilization, nuclear translocation and activation by a number of stimuli [32, 33]. Recent studies have indicated that Akt and its downstream mTOR could be act as the upstream signaling for Nrf2 Ser-40 phosphorylation [29, 34, 35]. On the other hand, Akt-mTOR inhibition was shown to block Nrf2 Ser-40 phosphorylation and subsequent activation [29, 34, 35]. In the current study, we showed that icariside II activated Akt-Nrf2 signaling in osteoblasts. More importantly, Akt inhibitor LY294002 almost blocked icariside II-induced Nrf2 accumulation and mRNA expression of Nrf2-dictated genes (*HO-1* and *NOQ-1*). Therefore, Akt is very likely the upstream signaling of Nrf2 activation by icariside II.

Besides being directly activated by the above ligands, EGFR may also be activated under a number of stimuli, the process known as “trans-activation” [36–40]. In the current study, we showed that EGFR-trans-activation could be a key upstream for icariside II-induced actions in osteoblasts. Treatment of icariside II induced significant HB-EGF production, which trans-activated surface EGFR in MC3T3-E1 cells. Blockage of EGFR trans-activation, via anti-HB-EGF, AG1478 (the EGFR inhibitor) or introducing the EGFR shRNA, almost abolished icariside II-induced Akt-Nrf2 activation in MC3T3-E1 cells. These results are consistent with recent finding showing that activation of EGFR can protect osteoblasts from Dex. For example, *Fan et al*., showed that activation of EGFR-Akt-mTOR signaling mediates epiregulin-induced osteoblast cytoprotection against Dex [10]. Similarly, the same group showed that pleiotrophin trans-activates EGFR and significantly rescues osteoblasts from Dex [18]. Further studies will be needed to explore how icariside II induces HB-EGF production and EGFR trans-activation. It would also be interesting to explore whether EGFR also mediates other downstream signaling by icariside II [11, 12].

## MATERIALS AND METHODS

### Chemicals and reagents

Icariside II, Dex and LY294002 were purchased from Sigma Chemicals (St. Louis, MO). Perifosine and MK-2206 were obtained from Selleck (Shanghai, China). All the antibodies of this study were purchased from Cell Signaling Technology (Danvers, MA).

### MC3T3-E1 cell culture

The culture and differentiation of the murine osteoblastic MC3T3-E1 cells were described in our previous studies [5–7].

### Culture of primary murine osteoblasts

In brief, the calvariae of neonatal mice was isolated, washed, and digested [5–7]. Digestions were neutralized, pooled, and filtered as described [5–7]. The resolving primary murine osteoblasts were cultured as reported [5–7]. All animals were maintained in accordance with the guidelines of the NIH. The protocol is approved by IACUC of authors’ institution.

### Cell survival assay

The Cell Counting Kit-8 (CCK-8, Dojindo Laboratories, Kumamoto, Japan) assay kit was utilized to test the survival of osteoblasts with indicated treatment [5–7].

### Cell apoptosis assay

The cell apoptosis histone-DNA ELISA plus kit (Roche, Palo Alto, CA) was utilized to quantify cell apoptosis in osteoblasts with indicated treatment, the detailed protocol was described previously [5–7].

### Cell death assay

As described in our previous studies [5–7], cell death was tested via medium release of lactate dehydrogenase (LDH), using a commercial available two-step LDH assay kit (Takara, Tokyo, Japan) [7].

### Western blots

Cellular protein lysates (30 μg per lane) were separated by the SDS-PAGE gels, and were transferred onto polyvinylidene difluoride (PVDF) membranes, which were blocked and incubated with indicated primary and secondary antibodies. Antibody-antigen binding was visualized though enhanced chemiluminescence (ECL, Pierce, Shanghai, China) reagents. The quantification of each Band was performed by the ImageJ software [5–7].

### shRNA

The two non-overlapping lentiviral shRNAs against murine Nrf2 were synthesized by Shanghai Genepharm Co. (Shanghai, China). The EGFR shRNA lentiviral particles were purchased from Santa Cruz Biotech (Shanghai, China). The lentiviral shRNA (10–15 μL/mL, per well) was added to cultured MC3T3-E1 cells (plated with 50% confluence) for 24 hours. Stable cells were selected by puromycin (0.5 μg/mL, Sigma) for 4–5 passages. Knockdown of targeted protein (EGFR or Nrf2) in the stable cells was confirmed by Western blot assay. The control MC3T3-E1 cells were infected with lentiviral scramble control shRNA (Santa Cruz).

### HB-EGF ELISA

After treatment of cells, the HB-EGF content in the conditional medium was tested by a commercial HB-EGF ELISA kit (Calbiochem, Suzhou, China)

### Reactive oxygen species (ROS) assay

ROS content in the cells was tested by a DCFH-DA fluorescent dye (Invitrogen, Shanghai, China). Briefly, after treatment, cells were incubated with 10 μM of DCFH-DA at room temperature for 30 min, which were and analyzed for fluorescence via a Fluorescence/Multi-Detection Microplate Reader (Synergy 2, BioTek, Winooski, VT). ROS intensity in the treatment group was expressed as fold change of that in the control group.

### RNA isolation and RT-PCR

Following treatment of cells, total cellular RNA was extracted through TRIzol reagents (Invitrogen, Shanghai, China). 500 ng of total RNA per sample was reverse-transcribed (RT) by the commercial RT-PCR kit (TOYOBO, Japan) according to the attached procedure. Real-time quantitative PCR (“RT-qPCR”) via SYBR green was performed in an ABI7300 machine (Applied Biosystems, Shanghai, China). The primers of Nrf2-associated genes, *Nrf2, HO-1* and *NQO1*, as well as *NADPH* were provided by Dr. Jiang's group [29, 34, 35]. 2^ΔΔCt^ was then calculated to yield fold expression relative to *GAPDH*.

### Statistical analysis

Experiments were repeated at least three times and consistent results were always obtained. *p* values < 0.05 (one-way ANOVA) were considered statistically significant.

## CONCLUSIONS

Collectively, we conclude that icariside II activates EGFR-Akt-Nrf2 signaling and protects osteoblasts from Dex. Icariside II might have translational value for treatment of Dex-associated osteoporosis and osteonecrosis.
